# Combining Classification with fMRI-Derived Complex Network Measures for Potential Neurodiagnostics

**DOI:** 10.1371/journal.pone.0062867

**Published:** 2013-05-06

**Authors:** Tomer Fekete, Meytal Wilf, Denis Rubin, Shimon Edelman, Rafael Malach, Lilianne R. Mujica-Parodi

**Affiliations:** 1 Department of Biomedical Engineering, State University of New York at Stony Brook, Stony Brook, New York, United States of America; 2 Department of Neurobiology, Weizmann Institute of Science, Rehovot, Israel; 3 Department of Psychology, Cornell University, Ithaca, New York, United States of America; Wake Forest School of Medicine, United States of America

## Abstract

Complex network analysis (CNA), a subset of graph theory, is an emerging approach to the analysis of functional connectivity in the brain, allowing quantitative assessment of network properties such as functional segregation, integration, resilience, and centrality. Here, we show how a classification framework complements complex network analysis by providing an efficient and objective means of selecting the best network model characterizing given functional connectivity data. We describe a novel kernel-sum learning approach, block diagonal optimization (BDopt), which can be applied to CNA features to single out graph-theoretic characteristics and/or anatomical regions of interest underlying discrimination, while mitigating problems of multiple comparisons. As a proof of concept for the method’s applicability to future neurodiagnostics, we apply BDopt classification to two resting state fMRI data sets: a trait (between-subjects) classification of patients with schizophrenia vs. controls, and a state (within-subjects) classification of wake vs. sleep, demonstrating powerful discriminant accuracy for the proposed framework.

## Introduction

Recent years have seen a growing interest in the analysis of functional connectivity [1] data resulting from brain mapping techniques such as fMRI. Complex network analysis (CNA), a subset of graph theory that focuses on the topologically complex networks often found in nature, has proved to be a powerful approach to quantifying important features of functional connectivity. These include general network properties such as its *functional segregation*, *integration*, *resilience,* and *centrality* [2], as well as quantifying the contribution of individual brain regions to the network at large.

CNA is performed on *graphs*, which topologically represent a matrix of functional connectivity. Functional connectivity can be derived from cross-correlation in different frequency bands, spectral coherence, or mutual information [3]–[10]. Normally, graph representations are obtained after excluding negative and auto-connections and are thresholded to retain only strongest connections, sometimes to the point of binarization.

Network properties can be defined globally, describing the structure of the entire network (e.g., *assortativity coefficient* [11], *closeness centrality* [12], *characteristic path length* [13], *clustering coefficient* [13], *global efficiency* [14], *graph transitivity* [15], *local efficiency* [14], *modularity* [16], *small-worldness* [17]). Or, they can be defined locally, on a per-node basis, so as to focus on neural regions of interest (e.g., *node betweenness centrality* [12], *node degree/strength*, *node characteristic path length* [13], *node clustering coefficient* [13] and *node global and local efficiency* [14].

Compared to voxel-based analyses used in more standard brain mapping, CNAs are theoretically parsimonious. However, *in practice*, the number of actual choices involved in CNA, such as the measure of connectivity, the connection threshold and frequency band chosen for the analysis, and the combinatorics associated with their optimization, could result in thousands of derived measures (see Figure S1). Thus, while CNA has the advantage of providing a large number of options by which functional connectivity can be probed, the diversity comes at the cost of having to find the “best” network model, with its concomitant multiple comparisons problem.

We propose that a natural and appealing means for reaping the full benefits of CNA is through combining complex network analysis with a learning procedure that seeks to optimize classification based on CNA features. By this we mean that the range of possible network measures derived from a given data set can be used as features for machine learning algorithms aiming at classifying functional data according to different subject groups or experimental conditions. The results of the procedure can then be assessed for significance without the need for correction for multiple comparisons. Moreover, as we describe below, feature selection incorporated into the classifier constitutes a clear-cut, readily interpretable, unbiased means of model selection.

Classification based on CNA measures holds appeal also for several other reasons. First, complex network analysis has proved to be sensitive in probing network features of psychiatric and neurological disorders [18]–[23]. Thus, incorporating CNA into the powerful framework of machine learning promises to be clinically applicable. Second, multivariate classification methods make it possible to explore how different measures of integration and segregation interact in characterizing complex brain conditions, including pathology.

Complex network measures can be naturally grouped into families: all global measures (both binary and weighted) derived from a single graph can be grouped together to what for simplicity’s sake we will refer to as a ***graph***. Similarly, local or per-node measures computed across different graphs can be grouped according to brain regions of interest, which we will refer to as an ***ROI***. We suggest that when applying machine learning to complex network measures, feature selection should be carried out by feature family; that is, by graph or ROI. In each case, model selection can be based on the performance of the feature families in question in supporting classification, that is, on discriminating ability between subject groups or experimental conditions. Once multivariate significance is established, the implicated ROIs/graphs can be further probed through post hoc analysis, enabling to focus on differences at a single-feature level to the degree they are present in the data.

The scenario in which features are fed to a classifier by families is commonly referred to as multiple kernel learning (MKL, [24]). In what follows, we describe a complete MKL optimization scheme incorporating graph/ROI selection: block diagonal optimization (BDopt). As a proof of concept, we describe the application of our scheme for complex network based classification to two resting state fMRI data sets designed to elicit trait (between-group; *N* = 18 patients with schizophrenia vs. controls) and state (within-group; *N* = 10 wake vs. sleep) differences, and show how CNA classification is not only useful in itself, but enables efficient exploration of the organization of functional architectures under various brain states.

## Methods

In this study we analyzed two resting state fMRI data sets, comparing *schizophrenia patients vs. controls*, as well as *wake vs. sleep*. Detailed information on data collection in experiment 2 is provided in Supplementary material S1. Formal definitions for all graph theoretic terms are provided in Supplementary material S2. This study was approved by the Institutional Review Board of the Weizmann institute of science; all participants provided informed written consent.

### 2.1 Data set 1– between-subjects Classification (Schizophrenic Patients vs. Controls)

#### 2.1.1 Study design

Data were acquired from a publicly available resting state fMRI dataset (http://hdl.handle.net/1926/1687). These data were collected and shared as per of the National Alliance for Medical Image Computing (NA-MIC) initiative, supported through the National Institutes of Health Roadmap for Medical Research, Grant U54 EB005149. Subjects were scanned using fMRI under resting state (closed-eyes) conditions, and included 18 males, of which eight were patients. There were no significant differences in age handedness or IQ (WIS) between patients and controls (see Table 1). Patients were medicated (all with atypical drugs, 1 with a conventional drug as well).

**Table 1:** Demographics of participants in study 1 (Schizophrenic Patients vs. Controls).

	age	handedness	IQ (WIS)
Patients	44.1±11.4	0.78±0.18	102.4±20.9
Controls	41.8±11.2	0.73±0.28	117.5±17.4
Significance	0.67	0.28	0.12594

Significance was assessed using two sided two sample *t*-tests.

#### 2.1.2 Image acquisition

fMRI scans were acquired using a 3T GE scanner at Brigham and Women's Hospital in Boston, MA, using an echo planar imaging (EPI). An eight-channel coil was used to perform parallel imaging using ASSET (GE) with a SENSE-factor  = 22. Runs were 10 minutes long, comprised 200 repetitions of a high resolution EPI scan (96×96 in plane, 3 mm thickness, TR-3000 ms, TE = 30, 39 slices).

#### 2.1.3 Image analysis

Standard preprocessing procedures were performed in SPM8 [25] including image realignment correction for head movements, normalization to standard 2×2×2 mm Montreal Neurological Institute space, and spatial smoothing with a 6-mm full width at half maximum Gaussian kernel. SPM movement estimates, as well as their squared magnitudes were regressed out of fMRI time series, as were the average time series from white matter, csf and global signal. Finally, the time series were detrended. Data for one patient exhibiting excessive movement were discarded.

### 2.2 Data set 2–Within-subjects Classification (Wake vs. Sleep)

#### 2.2.1 Study design

Ten healthy volunteers (4 female, ages 25.6±2) participated in this study and were scanned under two conditions: awake and asleep. For exact details see supplementary material S1.

#### 2.2.2 Image acquisition

The experiment was performed on a Siemens 3 Tesla Trio Magnetom MRI scanner, with birdcage radio frequency (RF) coil and a head only gradient coil designed for EPI. Functional images of blood oxygenation level dependent (BOLD) contrast comprising 46 axial slices were obtained with a T2*-weighted gradient echo EPI sequence (TR  = 3000 ms, TE  = 30, flip angle  = 90°, FOV 240 mm, matrix size 80×80, no gap 3×3×3 mm voxel, ACPC) covering the whole brain.

#### 2.2.3 Image analysis

Before analysis data were segmented into 180 TR sequences that were scored for sleep. Segments meeting the criteria for further analysis were preprocessed separately. Preprocessing procedures were performed in SPM8, including image realignment correction for head movements, normalization to a 3×3×3 mm Montreal Neurological Institute template, and spatial smoothing with a 8-mm full width at half maximum Gaussian kernel. The non-brain component of the time series, assessed by averaging the signal in the ventricles, was regressed out of fMRI time series [26], as were SPM movement estimates, and after which time series were detrended. Following the recommendation of a previous CNA study of sleep fMRI [27] we avoided regressing out the global signal in this case. Segments exhibiting excessive movement (>1 mm) were discarded. This resulted in 44 segments total (21 sleep), such that there was at least one example from each condition per subject.

### 2.3 Deriving Graphs from Imaging Data

The connectivity metrics chosen for this study were the correlation and partial correlation coefficients. We first extracted the average time series from the 116 automated anatomical labeling ROIs [28], which span the brain gray matter, using WFU pickAtlas [29]. The resulting time series were filtered into the 0.01–0.1 Hz [22] and 0.03–0.06 Hz frequency bands [30]. For each time series array - both the filtered and original time series - we computed the lagged correlations and partial correlations ranging from ±3TR and also derived the maximal correlation of the seven. Negative values were set to zero, as well as autocorrelations. The correlation matrices were thresholded to leave a fraction α of the strongest connections using 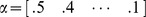 to produce 240 graphs (3×2×8×5; frequency bands, linear/partial correlation, seven lags and their maximum and five thresholds respectively - see Figure S1). From each resulting connectivity matrix both weighted and binary global features were harvested. For local measures, we focused on a subset of these graphs that has been reported to be discriminative, zero lagged partial correlations in the 0.01–0.1 Hz band [22], from which both binary and weighted features were derived for each ROI.

### 2.4 Complex Network Measures

The global measures employed in this study were: *assortativity coefficient* [11], *closeness centrality* [12], *characteristic path length* [13], *clustering coefficient* [13], *global efficiency* [14], *graph transitivity* [15], *local efficiency* [14], *modularity* [16], *small-world ratio*. The local measures [22] we utilized were: *node betweenness centrality* [12], *node degree/strength (the sum of edges)*, *node characteristic path length* [13], *node clustering coefficient* [13], and *node global and local efficiency* [14].

The reason we chose to use the small world ratio rather than small world index is twofold: for the binary ratio, normalization by the random small world ratio is vestigial - it is a mere multiplication by a constant that is factored out when features are normalized for scale before classification. In the weighted case, due to the exponentially greater combinatorial complexity, ratio estimates are associated with random variance introduced solely by permutation analysis, a clear artifact. We therefore applied normalization only during post-hoc analysis.

### 2.5 Motion Analysis

To compare head motion between groups five metrics were computed for each data point using the motion estimates resulting from SPM8's correction procedure. These metrics were later compared between groups using a *t*-test: 1) maximal displacement across *xyz* coordinates, 2) maximal degree shift 3) average translation 4) average rotation 5) frame displacement [31].

### 2.6 Classification

The goal of a classifier is to label data in a test set (e.g., patient vs. control, sleep vs. wake) according to information gleaned from learning data. All classification reported here was done using our NeuroClass (http://www.lcneuro.org/) - a publicly available Matlab toolbox for SVM based classification. NeuroClass utilizes the LIB-SVM toolbox [32] as its computational core.

#### 2.6.1 Feature scaling

Prior to analysis, each feature was normalized across subjects in the training sample via a *z* transform and the estimated mean and standard deviation were used to scale the test data. Normalization is required to avoid driving results due to trivial scale differences between features, and the necessity to adapt classifier parameters to scale.

#### 2.6.2 Multi kernel Block Diagonal optimization (BDopt)

Applying complex network analysis to neuroimaging data results in various features (i.e., measures), which in turn could simply be fed into a classifier. However, there are two substantial reasons for treating complex network measures as *families* of features rather than individual features. In the case of global measures, it is natural to treat various measures originating from a single graph as a single multidimensional representation, as different measures afford complementary information regarding basic properties of the dynamics: segregation, integration, centrality and resilience of the network [2]. In the case of local measures, grouping features according to ROI enables one to characterize a brain region in terms of its significance to the network as a whole, as well as to anatomically localize group differences in the case of pathology.

A general family of learning machines in which this can be naturally implemented is kernel sum machines, which is referred to as multiple kernel learning [MKL, 24]. In MKL, each family of features is used to derive a kernel matrix: 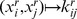 where 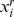 is the feature vector originating from the *r^th^* anatomical region (or graph) derived from the *i^th^* observation. Next, the kernels derived from all feature families are summed to produce a single kernel *K*, i.e.: 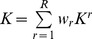. This kernel can then be fed to a kernel based learning machine such as a support vector machine (SVM).

**Figure 1:**
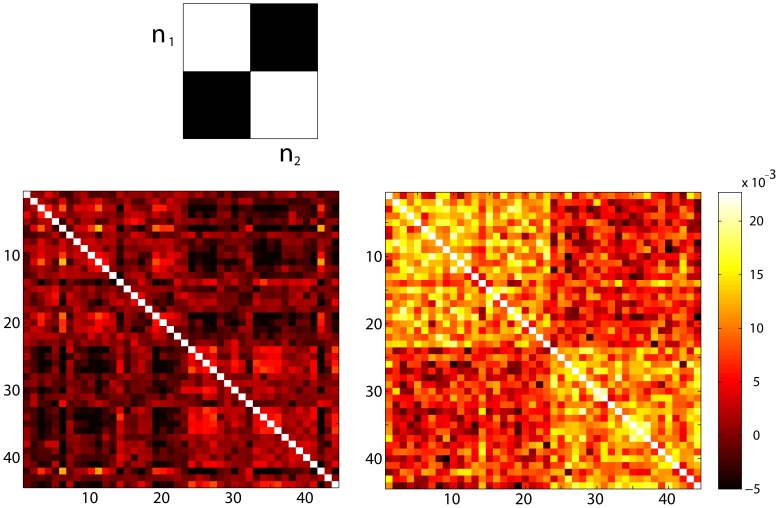
Kernel sum block diagonal optimization (BDopt). Top: An illustration of a block diagonal binary matrix; white = 1, in which *n_1_* and *n_2_* denote the sizes of groups 1 and 2 respectively. In case of an affinity matrix, structure such as in the above depiction represents an ideal scenario in which the ratio between inner group similarity to inter group similarity is maximal. Note that block diagonal binary matrices can represent any number of groups, and of course are not contingent on row/column ordering when used in our multi-kernel optimization routine. *Bottom:* We show an actual example derived from the *Wake vs. Sleep* data-set**,** during cross validation. Matrix entries are ordered by condition (awake, then sleep). *Left:* a direct kernel sum (all kernels normalized to unit diagonal). *Right:* a BDopt sum optimized according to the training sample labels. As can be seen, BDopt enhances block contrast (i.e. homogeneity within each of the four blocks).

We developed a novel optimization method for deriving optimal weights for feature families: multi kernel block diagonal optimization (BDopt). The kernel matrix can be thought of as representing the degree of similarity between feature vectors. In BDopt, the optimal weight vector *w* is found by maximizing the ratio of within-class to between-class similarity. In the ideal scenario – which would lead to perfect classification – the similarity within class would be maximal, e.g., attain some maximal value *s* for each pair of observations belonging to the group, while the similarity between instances belonging to different groups would be virtually zero. If the data are organized according to class, that is, first the examples belonging to the first class, followed by the examples from the second class and so on, then the resulting kernel matrix would have the form of a block diagonal matrix: the matrix entries corresponding to within class similarity would attain the value *s,* while all other values would be zero.(**Figure 1**). In general, this ideal similarity structure can be represented by a block-diagonal binary matrix. Thus *w_r_* can be found by minimizing the quadratic difference between the weighted sum of kernels and the block diagonal matrix *B*, i.e.: 
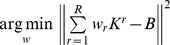
. This is, in fact, an ordinary least squares regression problem whose solution is given by 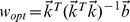, where 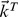 and 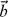 respectively denote the (column) vector representations of *K* and *B.* In the analysis described here, we applied BDopt to our data using a spherical kernel, i.e.: 
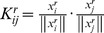
. The resulting SVM needs to be optimized for the soft margin parameter *C* to achieve optimal results as is always the case with SVM classification.

#### 2.6.3 Feature selection

In high dimensional classification, feature selection is crucial because increasing the dimensionality (number of features) leads to accumulation of not only signal, but also noise. Therefore, it is quite likely that for noisy features, the information they carry might be masked by noise as the number of features becomes excessive [33]. This is true both for the number of feature families (ROIs, graphs), and for the dimensionality of each family. BDopt readily allows for feature selection at both levels.

To select features within each family, we used the squared two-sample *t*-statistic [33], which allows the features to be ranked according to their discriminative power, given the training sample. After the features were ranked, only the top *k*% of the features in each family was retained. Note that this extends to multiclass scenarios in which the *F* statistic resulting from an ANOVA can be used.

To select feature families, recursive feature elimination (RFE, [34]), can be applied to the weighted kernels (i.e., RCK; [35]). In each iteration, an SVM is trained on the training sample, and the resulting weights are used to find the feature family that contributes the least to the classification. The quadratic norm of the SVM weights is given by 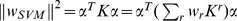. Given that the contribution of each weighted kernel to the SVM weights is 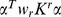, the least informative feature family is determined by 
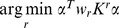
. This feature family can then be removed, and the process repeated. Note that this optimization results in a ranking of feature families according to their discriminative power.

#### 2.6.4 Cross-validation

Due to the small number of subjects in typical human neuroimaging studies, the most suitable choice for cross-validation (CV) seems to be a leave one out (LOO) scheme. In LOO, each subject in turn is removed from the pool. Next, classification is carried out on the remaining subjects (training data). Finally, the model is tested on the withheld subject (test) data, after which the test data is returned to the pool. The result for all subjects is averaged to yield a success rate.

In classification of neuroimaging time series, the dimensionality (number of features) greatly exceeds the number of data points, which can lead to overfitting the data. Overfitting can be circumvented by taking several measures. First, it is critical to carry out feature selection in conjunction with cross validation, i.e., independently for each training sample (the data after removing example *i*), otherwise the classification results will be biased, resulting in an inflated success rate. Second, regardless of the choice of kernel, SVMs have at least one hyper-parameter that has to be optimized for. Therefore, an additional CV step is necessary to find both the optimal number of feature families (i.e., ROIs or graphs) and the optimal SVM parameters. This means that for each of the LOO training sets, an additional LOO CV is carried out to select the abovementioned parameters. After they are computed, the model can be retrained on the full LOO training set, and tested on the withheld data. Given the small number of samples in a typical neuroimaging classification scenario, it is common for several parameter combinations to yield the maximal success rate, making the choice of a specific optimal combination of parameters arbitrary. We must, therefore, retain multiple versions of the trained classifier, corresponding to the multiple optimal combinations of parameters, and let the test outcomes be decided by a majority vote among those.

BDopt, and all the associated methods described herein were developed and tested on an independent data set of *N* = 33 schizophrenia patients and controls that we had previously collected [36]. Those data suggested that in general the spherical kernel appears to be a reasonable choice for classification, as it strikes a balance between nonlinear kernels, which have more parameters and are therefore more prone to overfitting, and the more robust but less discriminating linear kernel. BDopt classification as utilized in the present study is illustrated in Figure S2.

#### 2.6.5 Analysis of significance

Classification involving *k* pattern classes applied to a test set results in a contingency table (confusion matrix), that is, the frequency distribution *N^test^*∈(*N^test^_11_, N^test^_12_, …, N^test^_kk_*) of the patterns, where *N^test^_ij_* denotes the number of elements, belonging to class *i*, which were labeled by the classifier as members of class *j*. Three methods have been commonly used to assess the significance of such contingency tables [37]: Fisher’s exact probability test [38], the *χ^2^* test (which is a poor approximation when the sample size is small and contingency cells may have low counts), and Monte Carlo methods. While bootstrap methods are very appealing in this regard, due to the computational intensity of RCK they are not feasible. Accordingly, in the analysis we describe below we deployed the exact method.

#### 2.6.6 Post hoc analysis

If the performance of a classifier has proven to be significantly above chance, post hoc analysis can indicate which are the graph measures driving the result. On average (across CV folds), the performance of a classifier will peak at a certain number of feature families. Thus application of BDopt results in a unique subset of ranked graphs and ROIs. The CNA can be further probed by paired *t*-tests on individual measures, to assess the contribution of individual measures to the result.

If the number of contributing graphs or ROIs is substantial, it is possible to carry out post hoc analysis on measure families, rather than specific measures (e.g., the characteristic path length across different graphs and ROIs). One approach would be to analyze the distribution of the group comparison results for a given measure for the ROIs implicated by the classifier. Feature families that exhibited a higher fraction of significant comparisons than expected by chance are sought after. Under the null hypothesis, a single comparison is considered a random event generated by a binomial distribution with a parameter of 0.05. Thus *n* events (e.g. the comparisons across ROIs for a given measure) are can be assessed for significance with *B(n,0.05).* Additionally, the resulting *p* value is corrected for the number of features families. Further still, for each feature family, the fraction of significant pairwise comparisons for which the sign of the difference between groups was consistent can be calculated serving as a measure of the robustness of the result of the abovementioned analysis, and assessed for significance utilizing a binomial distribution with a parameter of 0.5, *B(n,0.5).* The resulting *p*-value is then corrected for the number of feature families. Thus, feature families that prove significant on both counts support group differences under a given network measure.

## Results

Both data sets were mined for global and local complex network measures. The CNA measures were then used as classification features. We compared the performance of several models - block diagonal optimization (BDopt), recursive composite kernels (RCK; [35]), recursive feature elimination (RFE, [34]), and standard SVM classification - on both global and local CNA features. As a control, classifier accuracy was compared to classification of the functional connectivity pattern used to derive local features - partial correlation in the 0.01–0.1 Hz frequency band - using BDopt. In all cases the spherical kernel model was applied and optimized for the soft margin parameter C = (2^−1^–2^6^), and initial feature selection, was according to a t^2^ threshold of 25%. All classification experiments were carried out using NeuroClass (http://www.lcneuro.org/) - a matlab toolbox for SVM based classification. To rule out confounds to the results caused by movement, we carried out two sample *t-*tests comparing maximal displacement in coordinate and angle, average translation and rotation, and frame displacement [31] between groups. No significant differences were found for either of the five metrics in both data sets: *p* = 0.29, 0.37, 0.41, 0.61,0.54, *df* = 43 for sleep vs. wake and *p* = 0.20, 0.28, 0.11, 0.098, 0.11, *df*  = 16 for schizophrenia vs. control.

### 3.1 Data Set 1 (Patients with Schizophrenia vs. Controls)

The results of the first classification experiment (patients vs. controls) using global CNA features are shown in **Table 2**. BDopt performed best achieving a success rate of 100%; Fisher’s exact probability test confirmed the results to be significant (*p = *0.00005). Success rate was higher than achieved by RCK and RFE, which both achieved 94% accuracy. In comparison, application of SVM with the same parameters did not yield significant classification.

**Table 2:** Classification accuracy of data set 1 (Schizophrenic Patients vs. Controls).

Classifier	accuracy	specificity	sensitivity	BalAcc	CV	significance
Bdopt	100	100	100	100	96	p<0.00005
RCK	94	90	100	95	96	p<5×10^–4^
RFE	94	90	100	95	95	p<5×10^–4^
SVM	65	80	43	61	69	p>0.1

Global complex network analysis (CNA) measures were derived for resting state data, and used as features for several support vector based classifiers. Significance was derived using Fisher's exact test. BalAcc denotes balanced accuracy. CV denotes accuracy across training folds.

**Figure 2:**
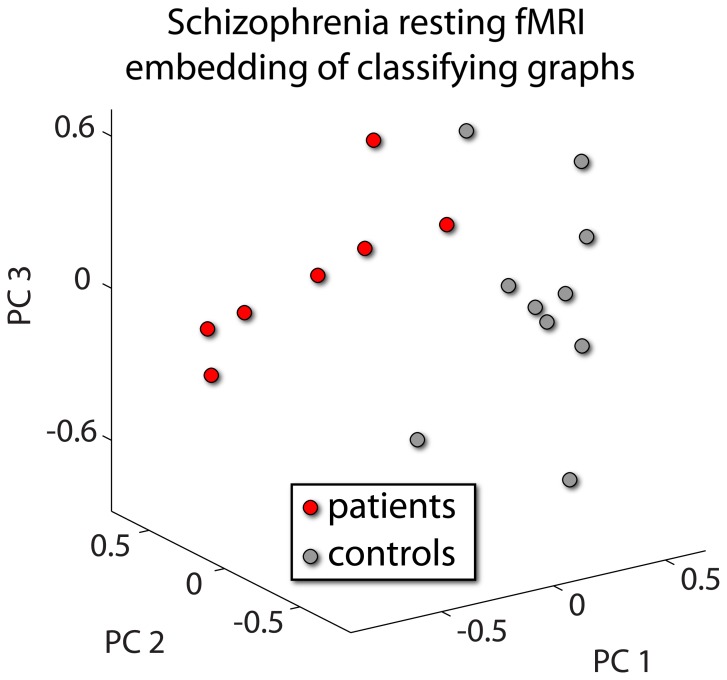
Post hoc analysis of *Patients with Schizophrenia vs. Controls*. Application of BDopt to these data resulted in a ranked list of 12 graphs. The global complex network measures in each of these 12 graphs were concatenated. A two sample *t-*test was applied after which only the top 25% of features were retained. Next, principal component analysis was carried out, and the loads of the data on the first three principal components were used to embed data in 3D.

**Figure 3:**
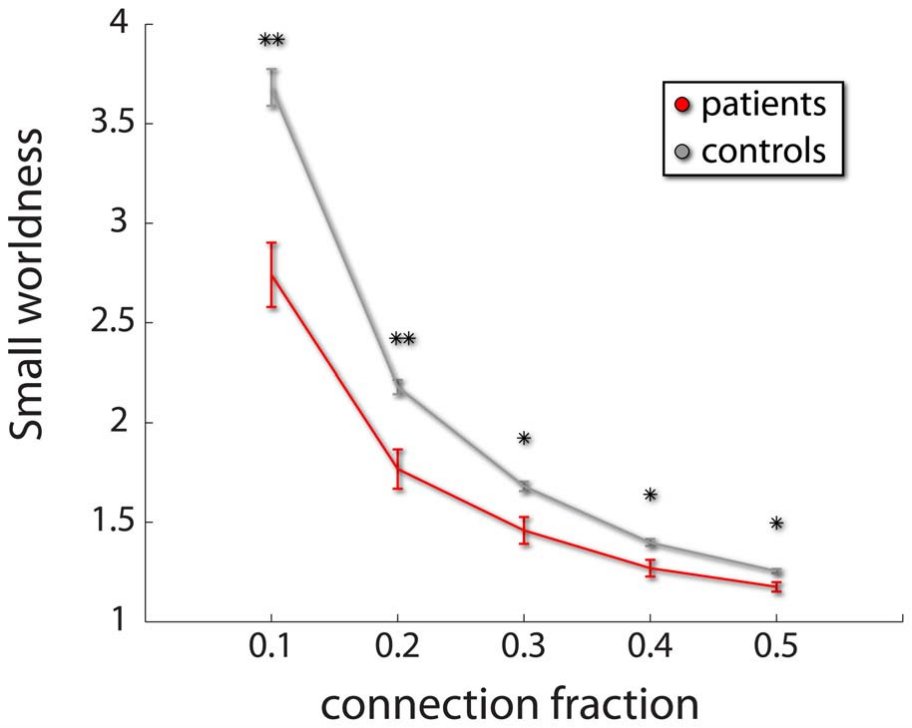
Difference in binary small worldness (*Patients with Schizophrenia vs. Controls*). Binary small worldness across the 5 levels of connectivity fraction threshold used in this study. The ratio was computed from the connectivity pattern from which the top ranked graph out of the 12 graphs selected by BDopt classification based on global CNA measures was derived. As reported previously by several studies, patients are characterized by a reduced small worldness indicating compromised network efficiency. * *p*<0.05 ** *p*<0.01 (uncorrected for number of graphs).

The average minimal number of graphs necessary to achieve perfect classification was 12. **Table 3** lists the top twelve graphs implicated by BDopt. We show a graphical representation of the discriminative power of the graphs selected by BDopt in **Figure 2**. Post hoc analysis could then be carried out on the individual measures across groups, by applying two-sample *t*-tests. In general, individual comparisons were moderately significant, and would not have survived correction for multiple comparisons. However, out of 216 (12 graphs×18 features) comparisons, 55 were significant (*p*<10^–23^ under a binomial test). Especially notable was the binary small world ratio that was significant in 6 out of the 12 graphs. As can be expected in schizophrenia fMRI [18]–[21], [39], in all cases patients exhibited a smaller ratio, indicating compromised efficiency of network topology (**Table 4**). The difference between patients and controls in the small world index is show in **Figure 3**.

**Table 3:** Graphs ranked by block diagonal optimization (*Patients with Schizophrenia vs. Controls*).

Correlation type	lag(TR)	connection fraction(%)
unfiltered	1	0.1
0.03–0.06 Hz	2	0.2
0.01–0.1 Hz	max	0.1
0.01–0.1 Hz	0	0.1
unfiltered	max	0.3
unfiltered	1	0.4
0.01–0.1 Hz partial	−1	0.1
0.01–0.1 Hz partial	−2	0.5
unfiltered	−1	0.1
unfiltered	1	0.3
0.03–0.06 Hz	2	0.4
0.03–0.06 Hz	3	0.1

We applied BDopt in conjunction with recursive kernel selection to global complex network measures. On average (across training data folds) accuracy peaked at 12 graphs. Graphs are listed according to their rank by the BDopt classifier.

**Table 4:** Difference in binary small world properties (*Patients with Schizophrenia vs. Controls*).

Group/graph	1	3	4	6	9	10
Patients	0.126±0.003	0.176±0.002	0.176±0.001	0.316±0.003	0.121±0.003	0.257±0.003
	(2.75±0.10)	(3.85±0.05)	(3.84±0.05)	(1.27±0.03)	(2.64±0.15)	(1.46±0.04)
Controls	0.169±0.003	0.188±0.002	0.187±0.001	0.349±0.003	0.168±0.003	0.296±0.003
	(3.70±0.07)	(4.10±0.05)	(4.09±0.03)	(.40±0.01)	3.67±0.06	(1.68±0.02)
Significance	P = 0.001	P = 0.038	P = 0.011	P = 0.021	P = 0.004	P = 0.015
[uncorrected]	(P = 0.001)	(P = 0.038)	(P = 0.011)	(P = 0.021)	(P = 0.004)	(P = 0.015)

Out of the 12 graphs selected by BDopt classification based on global CNA measures, six showed significant differences in the binary small world ratio between patients and controls, and in small worldness (as the difference is only in a scaling factor - shown in parenthesis). The numbers denoting the graphs correspond with Table 3. As reported previously by several studies, patients are characterized by a reduced small worldness index (and ratio), indicating compromised network efficiency.

In comparison, none of the applied models achieved significant successes employing local measures. This was true also for application of BDopt to the raw functional connectivity data. However, as post hoc analysis, we applied local analysis to the top connectivity pattern selected by BDopt global analysis - unfiltered 1 TR lagged correlations. The comparison of BDopt classification to the other classification methods, as well as classification based on raw connectivity data, is shown in **Table 5**. This time BDopt achieved moderate success (88% accuracy, *p = *0.002), while none of the other methods were significant.

**Table 5:** Classification using local features (*Patients with Schizophrenia vs. Controls*).

Classifier	accuracy	specificity	sensitivity	BalAcc	CV	significance
Bdopt	88	80	100	90	92	*P* = 0.002
RCK	76	90	57	74	91	*p*>0.1
SVM	71	90	43	67	79	*p*>0.1
RFE	68	65	71	68	79	*p*>0.1
FuncCon	56	60	43	52	74	*p*>0.1

Local complex network measures were computed for the top graph implicated by global CNA classification using BDopt (Table 3) - lag 1 (1 TR) correlation between the 116 ROIs spanning brain gray matter employed in this study. Significance was assessed using Fisher's exact test. FuncCon denotes BDopt classification using the raw functional connectivity. BalAcc denotes balanced accuracy. CV denotes accuracy across training folds.

### 3.2 Data Set 2 (Wake vs. Sleep)

As in experiment 1, data were mined for local and global complex network measures, and then classified according to state (wake vs. sleep). The results of local CNA based classification are shown in **Table 6**. Again BDopt attained the best accuracy - it yielded a success rate of 91% (95% sensitivity, 87% specificity, CV accuracy 90%). Fisher’s exact probability test showed this result to be significant (*n* = 44, *p*<2×10^–8^). In comparison, RCK yielded a success rate of 84%. Both RFE and BDopt classification of the raw functional connectivity data achieved 77% accuracy. As with Data Set 1, performance using an SVM with the same parameters on the features as a whole was not significant.

**Table 6:** Classifier accuracy for *Wake vs. Sleep- local CNA* features.

Classifier	accuracy	specificity	sensitivity	CV	significance
Bdopt	91	87	95	90	*p*<2×10^–8^
RCK	84	83	86	87	*p*<2×10^–5^
RFE	77	87	67	81	*p*<5×10^–4^
FuncCon	77	85	69	77	*p*<5×10^–4^
SVM	64	76	52	66	*p*>0.1

Five different classifiers were applied to local CNA features, derived from inter area partial correlations in the 0.01–01 Hz band. Classifiers are ordered according to accuracy. Significance was assessed using Fisher's exact test. CV denotes accuracy across training folds. FuncCon denotes BDopt classification using the raw functional connectivity.

**Figure 4:**
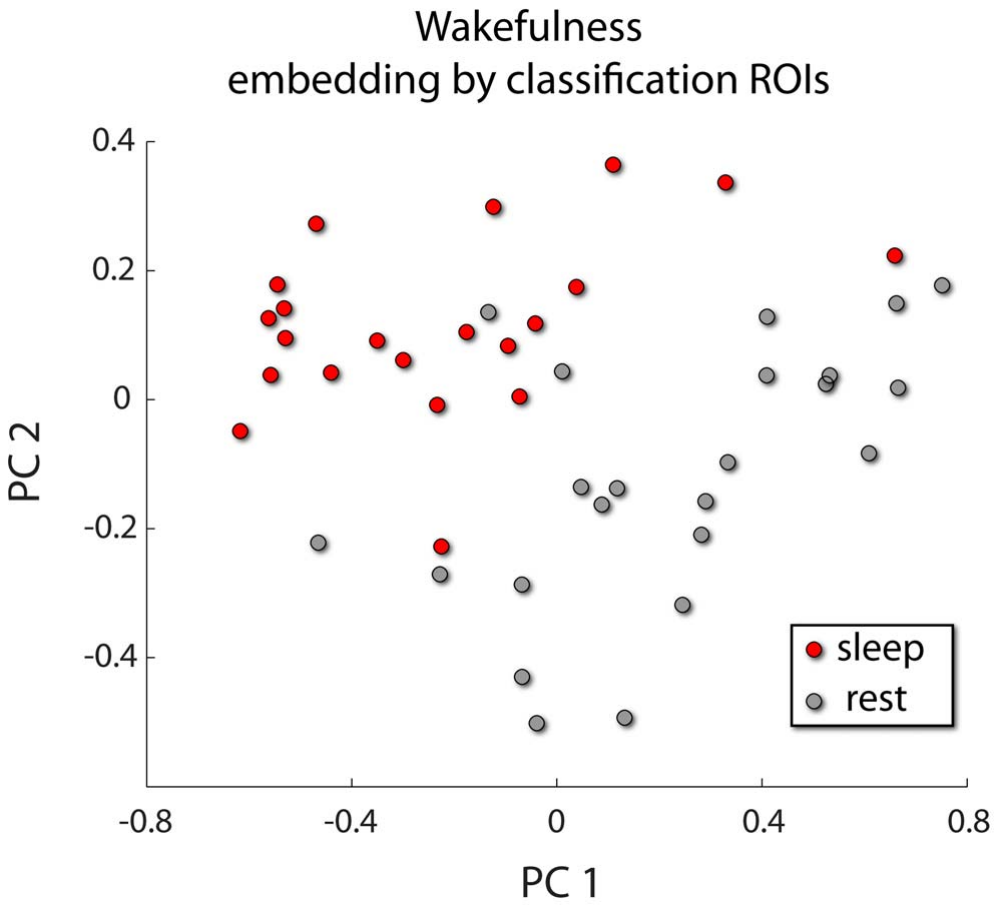
Post hoc analysis of *Wake vs. Sleep*. Application of BDopt to local complex network features resulted in a ranked subset of regions that led to maximal classification accuracy. A two sample *t-*test was carried out on the CNA features within these ROIs. The top 25% of features were retained and used for principal component analysis. These data were projected upon the first two principal components and color coded by condition.

**Figure 5:**
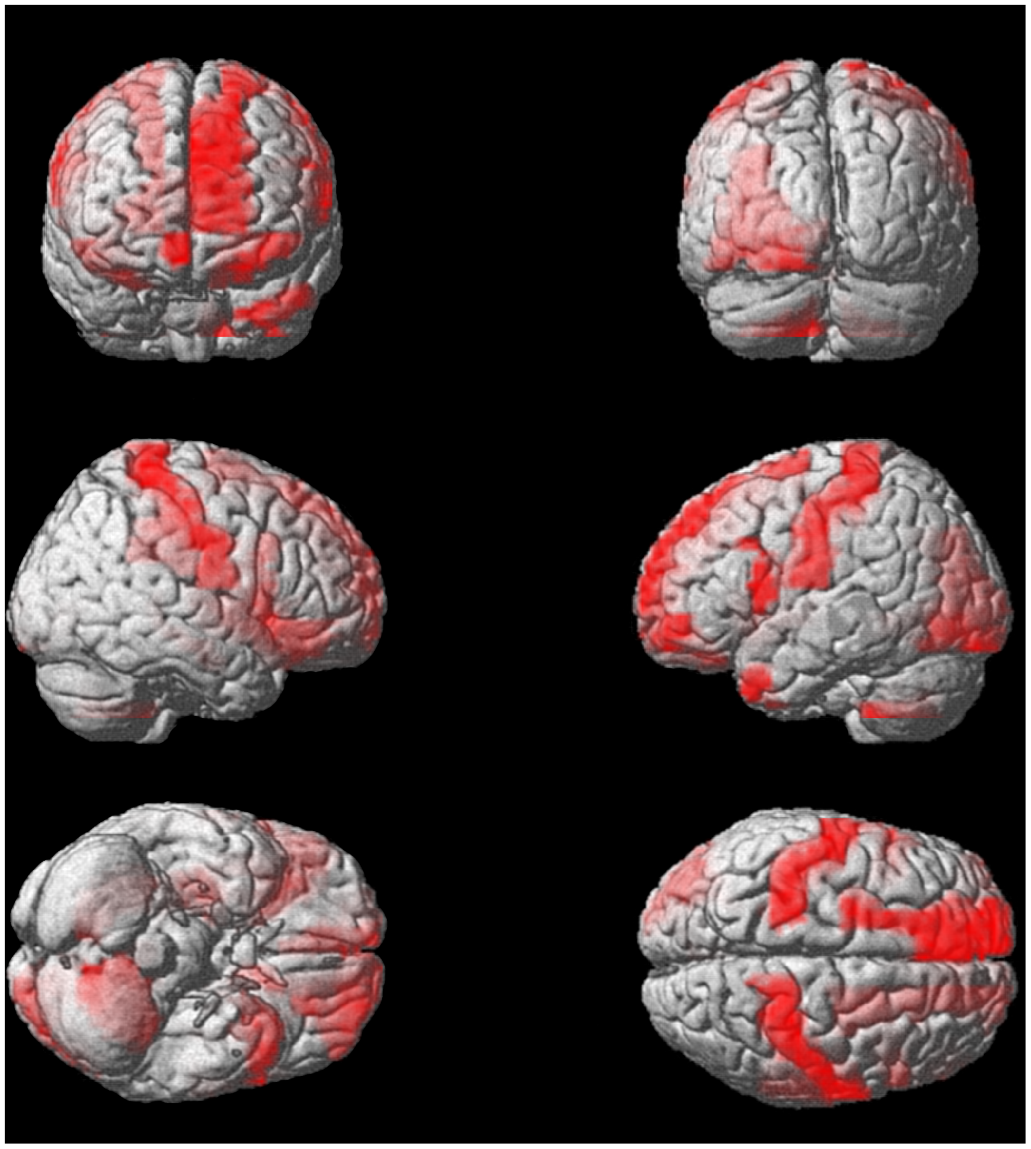
ROIs selected by BDopt for *Wake vs. Sleep*. Gray matter ROI participation (across training data folds) resulting from BDopt applied to local CNA measures. Only ROIs that contributed to maximal classification in a given training set are included.

Application of BDopt to local measures in conjunction with recursive kernel elimination [34], [35] resulted in a ranking of brain regions according to their contribution to successful classification. On average, classification peaked at approximately 18 brain areas, which are shown in **Table 7**. In **Figure 4**, we show a graphical representation of these regions’ discriminative power. **Figure 5** highlights the implicated ROIs according to their participation (the fraction of training data folds in which they were ranked among the first 18 regions).

**Table 7:** Anatomical ROIs selected through BDopt classification *Wake vs. Sleep.*

Anatomical ROI	participation	
Right insula	100%	
Left temporal pole: middle temporal gyrus	100%	
Left superior frontal gyrus, medial	100%	
Right superior frontal gyrus, medial orbital	100%	
Left caudate	100%	
Left postcentral gyrus	100%	
Left inferior frontal gyrus, opercular	100%	
Right putamen	100%	
Left superior frontal gyrus, dorsolateral	100%	
Right postcentral gyrus	100%	
Left Lingual gyrus	100%	
Left middle frontal gyrus, orbital	95%	
Right hippocampus	100%	
Vermis 3	84%	
Left Cerebelum 9	100%	
Vermis 1/2	98%	
Left Cerebelum 8	98%	
Right anterior cingulate	66%	

Local CNA features were used as the basis of classification. ROIs are listed according to their rank, achieved through recursive kernel elimination. *Participation* denotes the fraction of training folds in which a given ROI was selected during the classification.

We then conducted post hoc analyses on the ranked ROIs, in the form of paired *t*-tests for each measure/ROI conjunction across groups. In general, as in the analysis of our first data set, *p* values tended to be modest, and hence would not have survived correction for multiple comparisons for number of regions, let alone for the number of measures, models, and graphs tested. However, out of 1080 measures (18 ROIs×12 features×5 threshold values) 271 were significant using *p*<0.05. We then analyzed the distribution of *p*-values for a given measure. Four families of local measures showed significant excess of low *p*-values that was sign consistent across ROIs implicated by the classifier (see methods): binary and weighted node clustering coefficient and binary and weighted node local efficiency. This suggests that these were the primary features responsible for the classification, indicating low network efficiency during sleep.

In contrast global feature based classification achieved only moderate success, as summarized in **Table 8**.

**Table 8:** Classifier accuracy for *Wake vs. Sleep-* global CNA features.

Classifier	accuracy	specificity	sensitivity	CV	significance
RCK	78	85	71	81	P = 0.0004
BDopt	72	78	64	79	P = 0.01
RFE	61	61	61	68	p>0.1
SVM	52	61	43	61	p>0.1

Four different classifiers were applied to global CNA features, derived from inter area functional connectivity. Classifiers are ordered according to accuracy. Significance was assessed using Fisher's exact test. CV denotes accuracy across training folds.

## Discussion

Here we present a complete classification framework for conducting complex network analysis, permitting the flexibility afforded by various network measures, without the loss of power resulting from multiple comparisons. We show how a novel multiple kernel learning method, BDopt, through the process of finding the most discriminative combination of feature families (either connectivity patterns or brain regions), produces robust unbiased model selection. Combining both methods, the researcher can effectively mine functional brain imaging data for both global and local characteristics of the functional architecture at various scales.

BDopt achieved good classification accuracy when applied to global CNA measures derived from resting state data obtained from patients with schizophrenia and matched controls. Subsequent analysis showed that the result was driven to a large extent by the compromised small world network properties in schizophrenia. Our results are in line with previous results for resting state fMRI CNA in schizophrenia, which show that the illness produces marked differences in the global organization of functional connectivity, as measured by small-worldness and other indices of network efficiency [18]–[21]. Likewise, the results of local CNA-based classification of resting state fMRI under two distinct states of wakefulness suggest that, as expected based upon previous studies, there is a widespread reduction in network efficacy during reduced wakefulness [40]–[42].

In this study we chose to focus on the most used measure of functional connectivity - the correlation coefficient. However, there are several methodological questions associated with the use of correlation to estimate functional connectivity, which are reflected in the functional connectivity literature: The current understanding in fMRI is that functional connectivity is predominated by low frequency components [4]. Indeed here have been reports of successfully using different bandwidths for CNA such as the 0.01–0.1 Hz frequency band (e.g., [22]) and the 0.03–0.06 frequency band (e.g., [30]). This raises the question whether there should be a bandwidth of choice for analysis. Alternatively, it stands to reason that different bandwidths afford complementary information as to the underlying dynamics. Similarly, there have been reports of successful application of both linear and partial correlations (e.g., [43], [44] respectively), again raising the question if one should be preferred over the other. While it seems in order to factor out external influences when trying to gauge pairwise interactions (as in the use of partial correlations), given the limitations of regression as a denoising method, as well the fact that the number of ROIs typically analyzed is in the order of magnitude of the time points sampled, there is room for concern that important information might be thus lost. The latter concern is true for applying filtration methods to time series, due to the inherent limitations of filtration. Finally, although there have been reports of successful use of cross-correlation (lagged correlation) to study functional connectivity [45], recent simulation work [46] questions its validity in the context of fMRI derived functional connectivity. However for experimental modalities in which sampling rates greatly exceed fMRI modeling signaling delays might be critical for understanding the underlying dynamics [47].

One important advantage of the analysis framework we suggest, is that it does not necessitate an a priori commitment to seemingly competing analysis alternatives, but rather enables to answer such questions in a data driven unbiased way. Accordingly, in our study we derived complex network measures from several connectivity patterns: patterns resulting from the raw time series, the 0.01–0.1 Hz frequency and the 0.03–0.06 frequency band, from which we derived both linear and partial lagged correlations (cross-correlations −3TR to 3TR). Our results lend some support to the line of thought that these seemingly conflicting modes of analysis are complementary, at least to some extent. This might be expected given the multi-scale richness of the underlying dynamics.

Although in theory it is possible to optimize for both ROI and graph while carrying out local CNA classification if the 240 graphs we employed in our global analysis were utilized in local analysis, in practice it would not be viable: first, the number of features would have increased by two orders of magnitudes (the order of magnitude of regions in the AAL atlas), leading to compromised efficiency due to accumulation of noise resulting from the increased dimension [33]. Secondly, by the same token, if the optimization would be carried out on both ROIs and graphs, the sheer increase in feature family number, again two orders of magnitude, would result in unrealistic computation time. This might be circumvented to some extent by applying heuristics (e.g. halving the number of kernels in each iteration), albeit likely at the expense of accuracy.

The most straightforward way to handle this difficulty would be to carry out hierarchical classification: that is, begin with global analysis to single out a small subset of putative graphs that could be explored using local analysis (provided a significant classification result). However in the two data sets we report here it was not the case that significant global differences necessitate significant local differences, and vice versa. This raises the (unsurprising) possibility that some states of the brain indeed have global signatures, while others are contained mainly to local networks.

Alternatively, it might be possible to carry out optimization on both ROIs and graphs by conducting an elaborated process of cross validation in which half of the training data are used to optimize for ROI, and the remaining training data to optimize for graphs. Of course for such a hybrid scheme to be meaningful the amount of data available for analysis would have to exceed the sample sizes typical in neuroimaging.

Several studies have combined network measures and learning algorithms: In one study [47], five local measures were computed from 36 ROIs. A classifier combined with feature selection was applied optimizing for connection fraction threshold. Another study [48] applied linear discriminant analysis to global CNA measures, derived from anatomical connectivity graphs originating from a mouse model for neurological disease and control mice. This resulted in an 18 dimensional space, clearly a different scenario from the one we describe. In contrast BDopt outperformed two benchmark classifiers - RFE and kernel averaging, and further still, allows for hierarchical inference, first at the level of the entire feature set, then at the level of feature families and finally at the single feature level. Another recent study [49] also indicates the promise in combining CNA and SVM classification.

In summary, multiple kernel methods, such as our BDopt, seem to be a natural framework for conducting complex network analysis: they allow to gauge network properties both globally and locally, they offer the benefits of multivariate methods both in powerful inference and model selection yet retain interpretability due to their hierarchical nature, and finally, our results suggest that CNA based MKL might hold promise for application in clinical settings.

## Supporting Information

Figure S1 **CNA and feature number – global analysis.** Many outstanding questions remain regarding the application of complex network analysis to brain circuits necessitating exploratory analysis. Accordingly, effective means of model selection are called for. Similarly, given the complex multi-scale nature of the CNS, it is desirable to employ multi-scale models to neuronal time series, as well as allow degrees of freedom to capture temporal facets of interactions resulting from communication delays. Graph selection via classification can then help resolve to what extent such phenomena are prevalent in given data. In the above example, the proliferation of features resulting from exploratory analysis is illustrated. Note that local analysis increases feature number by at least two orders of magnitude.
(PDF)

Figure S2 **An illustration of a BDopt classification experiment.**
(PDF)

Supplementary Material S1 **Data set 2– Wake vs. Sleep.**
(DOCX)

Supplementary Material S2 **Complex Network Measures.**
(DOCX)
