# Supplementary material for: Combining Classification with fMRI-Derived Complex Network Measures for Potential Neurodiagnostics
Source: PLoS One. 2013 May 6;8(5):e62867. doi: 10.1371/journal.pone.0062867 (PMC3646016; doi:10.1371/journal.pone.0062867)
Supplement: Figure S1 — CNA and feature number – global analysis. Many outstanding questions remain regarding the application of complex network analysis to brain circuits necessitating exploratory analysis. Accordingly, effective means of model selection are called for. Similarly, given the complex multi-scale nature of the CNS, it is desirable to employ multi-scale models to neuronal time series, as well as allow degrees of freedom to capture temporal facets of interactions resulting from communication delays. Graph selection via classification can then help resolve to what extent such phenomena are prevalent in given data. In the above example, the proliferation of features resulting from exploratory analysis is illustrated. Note that local analysis increases feature number by at least two orders of magnitude. (PDF) [file pone.0062867.s001.pdf]

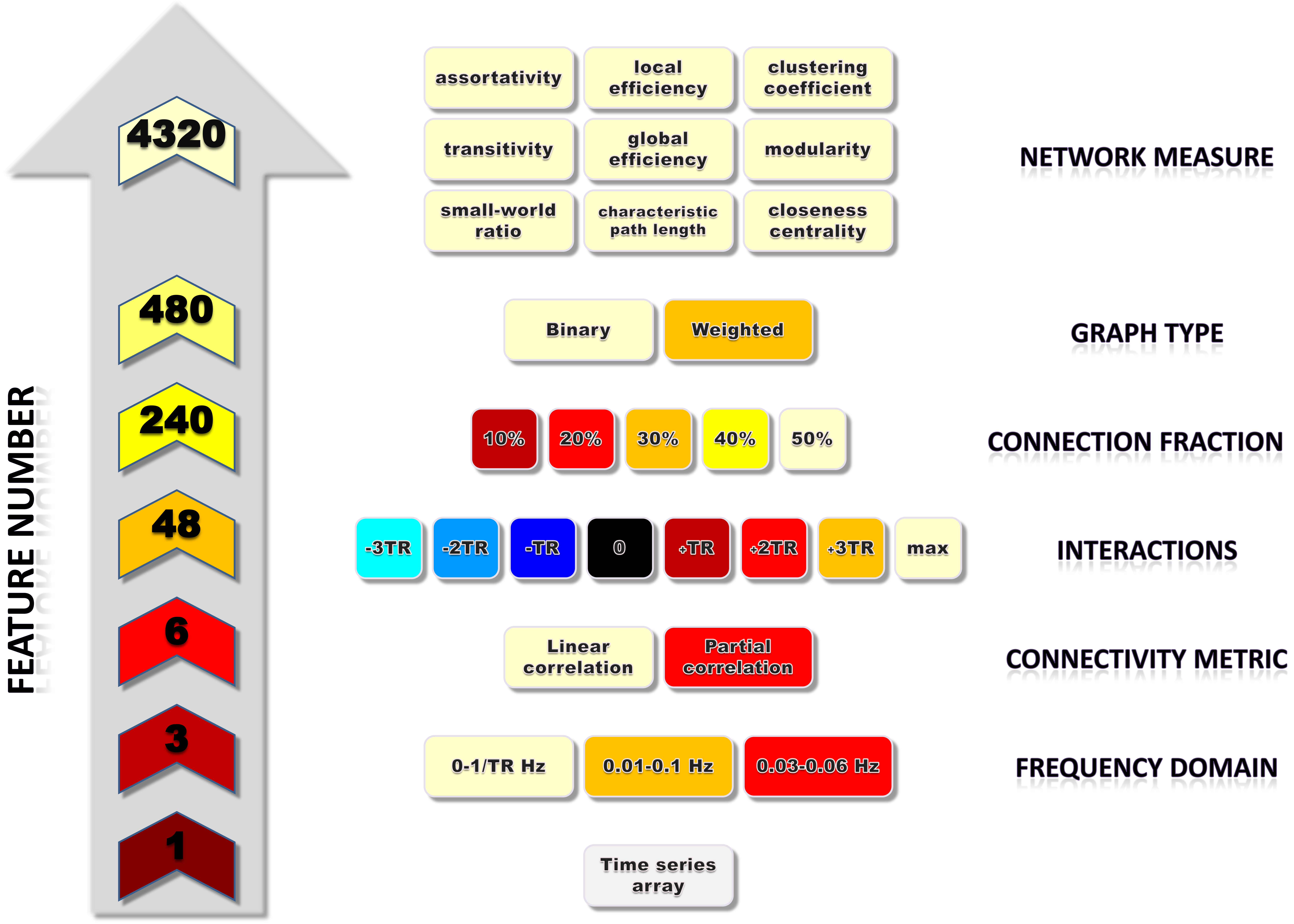

FEATURE NUMBER
4320
480
240
48
6
3
1
assortativity
local efficiency
clustering coefficient
transitivity
global efficiency
modularity
small-world ratio
characteristic path length
closeness centrality
NETWORK MEASURE
Binary
Weighted
GRAPH TYPE
10%
20%
30%
40%
50%
CONNECTION FRACTION
-3TR
-2TR
-TR
0
+TR
+2TR
+3TR
max
INTERACTIONS
Linear correlation
Partial correlation
CONNECTIVITY METRIC
0-1/TR Hz
0.01-0.1 Hz
0.03-0.06 Hz
FREQUENCY DOMAIN
Time series array
