# Supplementary material for: Combining Classification with fMRI-Derived Complex Network Measures for Potential Neurodiagnostics
Source: PLoS One. 2013 May 6;8(5):e62867. doi: 10.1371/journal.pone.0062867 (PMC3646016; doi:10.1371/journal.pone.0062867)
Supplement: Figure S2 — An illustration of a BDopt classification experiment. (PDF) [file pone.0062867.s002.pdf]

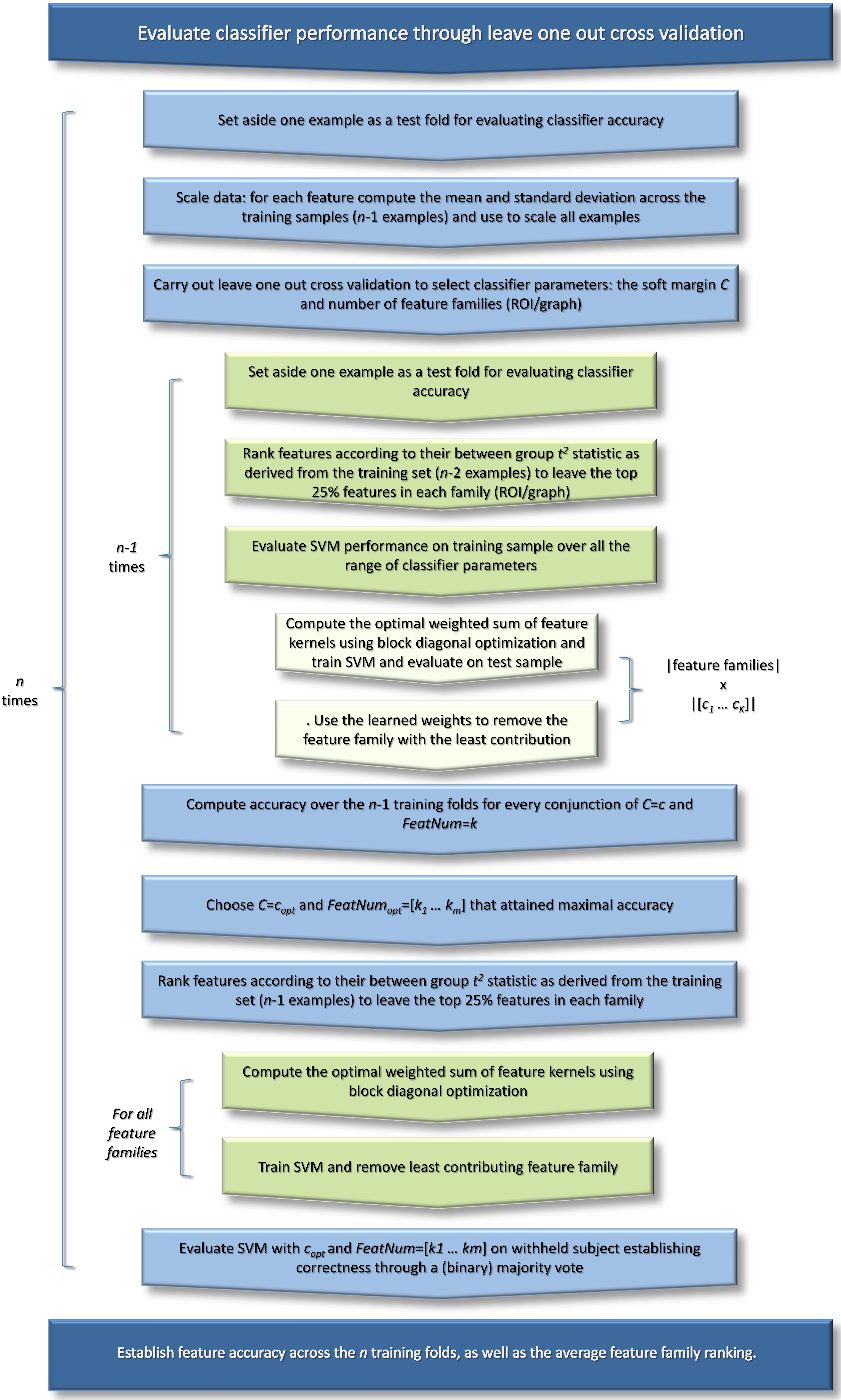
Evaluate classifier performance through leave one out cross validation
Set aside one example as a test fold for evaluating classifier accuracy
Scale data: for each feature compute the mean and standard deviation across the training samples (*n*-1 examples) and use to scale all examples
Carry out leave one out cross validation to select classifier parameters: the soft margin *C* and number of feature families (ROI/graph)
Set aside one example as a test fold for evaluating classifier accuracy
Rank features according to their between group t^2 statistic as derived from the training set (*n*-2 examples) to leave the top 25% features in each family (ROI/graph)
Evaluate SVM performance on training sample over all the range of classifier parameters
Compute the optimal weighted sum of feature kernels using block diagonal optimization and train SVM and evaluate on test sample
. Use the learned weights to remove the feature family with the least contribution
|feature families|
x
$|[c_1 \dots c_K]|$
n-1
times
n
times
Compute accuracy over the *n*-1 training folds for every conjunction of *C*=*c* and *FeatNum*=*k*
Choose $C=c_{opt}$ and $FeatNum_{opt}=[k_1 \dots k_m]$ that attained maximal accuracy
Rank features according to their between group t^2 statistic as derived from the training set (*n*-1 examples) to leave the top 25% features in each family
Compute the optimal weighted sum of feature kernels using block diagonal optimization
Train SVM and remove least contributing feature family
For all feature families
Evaluate SVM with c_{opt} and *FeatNum*=[*k1* … *km*] on withheld subject establishing correctness through a (binary) majority vote
Establish feature accuracy across the *n* training folds, as well as the average feature family ranking.
