# Supplementary material for: Combining Classification with fMRI-Derived Complex Network Measures for Potential Neurodiagnostics
Source: PLoS One. 2013 May 6;8(5):e62867. doi: 10.1371/journal.pone.0062867 (PMC3646016; doi:10.1371/journal.pone.0062867)
Supplement: Supplementary Material S1 — Data set 2– Wake vs. Sleep. (DOCX) [file pone.0062867.s003.docx]

Supplementary material S1: *Data set 2 – Wake vs. Sleep*

*Study Design:* Ten healthy volunteers (4 female, ages 25.6±2) participated in this study and were scanned under two conditions: awake and asleep. All subjects spent two consecutive nights inside the MRI scanner, without prior sleep deprivation in order to achieve sleep as naturally as possible. On the experimental nights, subjects fell asleep in a room outside the scanner at around 1:00 a.m. After one hour of sleep, subjects were woken up and escorted to the MRI scanner. Subjects' head were placed on a foam cushion for stabilization, and MR compatible earphones (MR ConFon, Magdeburg, Germany) that substantially reduce external noise were placed on the ears. The subjects were then scanned for three hours every night (2:30 – 5:30 a.m.), or until they felt uncomfortable (on average subjects were scanned for five and a half hours). During the course of the scan an objective physiological measure of sleep (either EEG or pulse) was acquired. The participants were requested to remain with their eyes closed throughout the experiment, and to immediately report if they awoke from a dream in the middle of the scan. No subject reported a dream. During these three-hour sessions, subjects passively listened to auditory stimuli of nine minutes length. Each nine-minute run contained auditory stimuli played at a fixed comfortable non-arousing volume, but sufficiently loud so the stimuli were heard clearly over the scanner noise. Auditory runs were interspersed with nine-minute runs of silent rest. Here we report the analysis of the silent rest runs.

*EEG Acquisition and Analysis* Three of the subjects underwent simultaneous polysomnographic recording throughout the experimental nights to assess sleep staging. The EEG caps included four scalp electrodes, referenced online to FCz, as well as two horizontal electrooculogram (EOG) channels, two vertical EOG channels, two electromyography (EMG) channels, and one electrocardiogram (ECG) channel. Abrasive electrode gel (ABRALYT 2000) was used to keep electrode-skin impedance at under 10k ohm, in addition to the 5/15k ohm resistor built into the electrodes (5k ohm for scalp electrodes, 15k ohm for EOG, EMG and ECG electrodes). EEG was recorded in the scanner room simultaneously with fMRI acquisition by using a magnetic resonance (MR) compatible 32-channel amplifier (BrainAmp MR plus; Brain Products) and an MR compatible EEG cap (BrainCapMR; FalkMinowServices) with 11 pin type electrodes. Data were transferred through fiber optic cables to a personal computer, in which the EEG system running Vision Recorder Software, version 1.10 (Brain Products) was synchronized to the scanner clock. The EEG was digitized at a 5000 Hz sampling rate with 500 nV resolution. Data were analog-filtered by a band-limiting low-pass filter at 250 Hz (30 dB per octave) and a high-pass filter with a 10 second time constant corresponding to a high-pass frequency of 0.0159 Hz. Scanner gradient artifacts were removed relative to the MR volume gradient artifact onset, which was indicated by a trigger received from the MR system and recorded with the EEG, as implemented in Analyzer 1.05 (Brainproducts, Munich. Germany). Ballisto-cardiographic artifacts were removed by using the EEGLAB plug-in FMRIB 1.0 (Center for Functional MRI of the Brain, Oxford, UK). Based on the identified heartbeat events, an artifact template was defined as the median across a sliding window of 30 heartbeats centered on the heartbeat event being processed. Data from the scalp electrodes were band-passed for delta (1-4 Hz) and beta (21-23 Hz) rhythms. The momentary ratio of the beta power to delta power was calculated for later sleep scoring assessment, (“sleep ratio”). In addition, the heart rate was calculated from the ECG channel using the EEGLAB plug-in for heartbeat detection, to validate the use of HR alone to identify sleep.

*Heart Rate Recording and Analysis*: To alleviate subject discomfort under the restricting conditions of the MRI scanner, the remaining subjects were monitored for sleep only by a pulse electrode attached to their left index finger (TSD110-MRI, Biopac Systems, Inc, Coleta, CA). We measured pulse using an MRI compatible pressure pad and the signal was conducted outside the scanner room by plastic tubes into a transducer amplifier located in the next room (DA100C, Biopac Systems, Inc, Coleta, CA). The recording commenced with a trigger delivered from the scanner at the beginning of the session.

*Validation of Sleep from Heart Rate via EEG*: The subjects' state (asleep vs. awake) in each scan during the experimental nights was evaluated offline by three experienced scorers (M.W. M.R. and A.H.), either according to the scalp EEG sleep ratio (beta/delta power) graph or according to the pulse rate. When scoring according to EEG data, scans with low sleep ratio, indicating a decrease in high frequencies and increase in delta rhythm, were classified as sleep [[1](#_ENREF_1),[2](#_ENREF_2)]. Elevations in the ratio graph were scored as awakenings. Additionally, a manual score of the EEG trace was conducted. No scan was classified as REM sleep according to standard criteria for the subjects staged according to EEG. The sleep ratio and the ECG heart rate data showed a clear correlation in accordance with previous studies [[3](#_ENREF_3),[4](#_ENREF_4)]. This implied that the pulse rate by itself could serve as a measure of wakefulness. To test this possibility, sleep scoring (five categories in increasing order – sleep, sleep/mixed-state, awake/mixed-state, awake) based on two subjects was done independently, yielding a correlation of *r*=.92. Therefore, characterization for sleep vs. awake states for the remaining N=8 subjects was done according to heart rate, in which periods of increasing pulse were classified as awakenings, and a descent in heart rate indicated sleep [[3](#_ENREF_3)]. For the subjects for whom only pulse was acquired, no attempt was made to differentiate between REM and NREM sleep stages. Runs that included both wakefulness and sleep according to the scoring were discarded from the analysis: only runs identified as fully asleep or fully awake were used in the analysis. After exclusion of motion contaminated frames a total of *n*=44 segments originating from all patients remained.

1. Rechtschaffen A, Kales A A manual of standardized terminology, techniques and scoring system for sleep stages of human subjects. 1968 Brain Information Service. Los Angeles, CA.

2. Carskadon MA, Dement WC (2000) Normal human sleep: an overview. Principles and practice of sleep medicine 4: 13-23.

3. Penzel T, Kantelhardt JW, Grote L, Peter JH, Bunde A (2003) Comparison of detrended fluctuation analysis and spectral analysis for heart rate variability in sleep and sleep apnea. IEEE Trans Biomed Eng 50: 1143-1151.

4. Devot S, Bianchi AM, Naujokat E, Mendez MO, Braurs A, et al. (2007) Sleep monitoring through a textile recording system. Conf Proc IEEE Eng Med Biol Soc 2007: 2560-2563.
