# Supplementary material for: Combining Classification with fMRI-Derived Complex Network Measures for Potential Neurodiagnostics
Source: PLoS One. 2013 May 6;8(5):e62867. doi: 10.1371/journal.pone.0062867 (PMC3646016; doi:10.1371/journal.pone.0062867)
Supplement: Supplementary Material S2 — Complex Network Measures. (DOCX) [file pone.0062867.s004.docx]

Supplementary material S2: Complex Network Measures

**Basic definitions:** Let $N=\left\{\begin{matrix} 1, & 2, & \ldots& n \end{matrix}\right\}$ be the set of all nodes in the graph, and *v_ij_* the weight of the edge connecting nodes *i*, *j*. The degree of node *i* is $\kappa_{i}=\sum_{j} v_{ij}$. The shortest path distance is: $D_{ij}=\sum_{v_{kl}\in g_{i\leftrightarrow j}} f(v_{kl})$where $g_{i\leftrightarrow j}$is the shortest path between *i* and *j*, and *f* is a map from weight to length (e.g., the reciprocal function for correlation matrices, where higher values indicate shorter effective distances; or the identity function in case of a binary graph).

**Characteristic path length** [[1](#_ENREF_1)] of a graph is given by: where is the **node** **characteristic path length**

**Global efficiency** [[2](#_ENREF_2)] of a graph is: where is the **node** **global efficiency**

**Clustering coefficient** [[1](#_ENREF_1)] is: where is **node** **clustering coefficient** and

**Transitivity of a graph** [[3](#_ENREF_3)]: $T=\frac{\sum_{i} 2t_{i}}{\sum_{i} \kappa_{i}(\kappa_{i}-1)}$

**Local efficiency** [[2](#_ENREF_2)] of a graph: where $E_{i}=\frac{\sum_{j\neq i,h} \left(v_{ih}v_{ij}\left[d_{hj}(N_{i})\right]^{-1}\right)^{\frac{1}{3}}}{\kappa_{i}(\kappa_{i}-1)}$ is the **node local efficiency** and $d_{hj}(N_{i})$is the length of the shortest path between *h* and *j*, which contains only neighbors of *i*.

**Closeness centrality** [[4](#_ENREF_4)]:

**Node betweenness centrality** [[4](#_ENREF_4)]: where is the number of shortest paths between *h* and *j*, and is the number of shortest paths between *h* and *j* that pass through *i*.

**Assortativity coefficient** [[5](#_ENREF_5)]: where $l^{v}=\sum_{i,j} v_{ij}$

**Small world ratio** [[6](#_ENREF_6)]: $S=\frac{C}{L}$. To compute the related small worldness index, the respective quantities are*C_r_* and *L_r_* are computed for random graphs arising from permuting the original connectivity patterns and then computing the ratio between *S* and *S_r_*.

**Modularity** [[7](#_ENREF_7)]: where *m_i_* is the module containing node *i*, and $\delta_{m_{i}m_{j}}$= 1 if *m_i_*=*m_j_* and 0 otherwise.

1. Watts DJ, Strogatz SH (1998) Collective dynamics of small-world. Nature 393: 440-442.

2. Latora V, Marchiori M (2001) Efficient behavior of small-world networks. Physical Review Letters 87: 198701.

3. Newman MEJ (2003) The structure and function of complex networks. SIAM review 45: 167-256.

4. Freeman LC (1979) Centrality in social networks conceptual clarification. Social networks 1: 215-239.

5. Leung C, Chau H (2007) Weighted assortative and disassortative networks model. Physica A: Statistical Mechanics and its Applications 378: 591-602.

6. Humphries MD, Gurney K (2008) Network ‘small-world-ness’: a quantitative method for determining canonical network equivalence. PLoS One 3: e0002051.

7. Newman MEJ (2004) Fast algorithm for detecting community structure in networks. Physical Review E 69: 066133.
